# Family Self-management Program for Hypertension Management and Sodium Consumption Adherence: A Parallel Randomized Control Trial Among Family Caregivers and People With Hypertension

**DOI:** 10.34172/jrhs.2024.163

**Published:** 2024-09-30

**Authors:** Tantut Susanto, Sri Hernawati, Rismawan Adi Yunanto, Ira Rahmawati, Niken Asih Laras Ati, Wahyuni Fauziah

**Affiliations:** ^1^Department of Community, Family and Geriatric Nursing, Faculty of Nursing, Universitas Jember, Jember, Indonesia; ^2^Department of Oral Disease, Faculty of Dentistry, Universitas Jember, Jember, Indonesia; ^3^Department of Emergency and Critical Nursing, Faculty of Nursing, Universitas Jember, Jember, Indonesia; ^4^Department Maternal and Pediatric Nursing, Faculty of Nursing, Universitas Jember, Jember, Indonesia; ^5^Department of Neurology, Dr. H Koesnadi Regional Hospital of Bondowoso, Bondowoso, Indonesia

**Keywords:** Family self-management programs, Hypertension, Caregivers, Blood pressure, Sodium

## Abstract

**Background:** Hypertension (HTN) has become a serious health problem in developing countries. The family has an important role in maintaining blood pressure (BP) at home, and sodium diet compliance in people with HTN needs to be developed. Therefore, this research aimed to evaluate the effectiveness of a family self-management program (FSMP) in HTN management and compliance with sodium consumption in rural areas of Indonesia.

**Study Design:** A parallel-group, single-blind randomized controlled trial.

**Methods:** One hundred twenty-six eligible participants were randomly allocated to an intervention (n=63) and a control group (n=63). Participants in the intervention group received a 24-week (6-month) FSMP intervention. The primary outcome measures included the systolic and diastolic BP, the Score Sodium Questionnaire (SSQ), and the Morisky Medication Adherence Scale 8 (MMAS-8). The Knowledge of Health Care for HTN questionnaire and the Efficacy and Behavior Toward Health Care for Patients with HTN questionnaire were used to assess the secondary outcome.

**Results:** The final results were analyzed from 121 participants (n=61 intervention and n=60 control group). The repeated analysis of variance (ANOVA) test results demonstrated significant effects on the management of HTN and adherence to sodium consumption as indicated by systolic (*P*=0.004) and diastolic BP (*P*=0.006), SSQ (*P*<0.001), MMAS 8 (*P*<0.001), caregivers’ knowledge (*P*<0.001), caregivers’ self-efficacy (*P*<0.001), and caregivers’ behaviors (*P*=0.005).

**Conclusion:** The FMSP emerges as a promising strategy for managing BP and adherence to sodium consumption in people with HTN through the support of family caregivers and selfmanagement activities.

## Background

 Non-communicable diseases (NCDs) account for around 41 million, or 74% of, deaths worldwide; one of them is hypertension (HTN),^1^ which is still a health problem in developing countries.^2^ The prevalence of HTN in Indonesia has reached 34.11%, and in East Java province, it was slightly higher than the national prevalence of HTN (36.32%).^3^ Indonesia is known as an agricultural country, and agricultural communities are communities that have a high risk of experiencing health problems,^4^ including HTN.^5^ Based on our previous research, in agricultural areas, there was a percentage of pre-systolic HTN (20.1%), grade 1 and 2 systolic HTN (25.1%), and grade 1 and 2 diastolic HTN (35.8%).^6^ The findings of previous research on people with HTN in rural areas showed an increased risk of HTN, one of which is related to high salt intake, including knowledge, consumption, and dietary habits of salt.^7,8^ The high incidence of HTN, which is an NCD problem, requires treatment management with different strategies.

 Many factors affect the management of HTN in people. The management of HTN depends on sociodemographic status, such as culture,^9^ economic status,^10^ gender, education,^11^ and eating habits.^12^ However, in managing HTN, awareness, knowledge, attitudes, and behavior of people with HTN regarding the disease play an important role. Several barriers were found to be related to uncontrolled HTN management, including knowledge and awareness of people with HTN regarding HTN treatment, reducing salt intake, weight loss, and regular physical activity, which are ultimately healthy lifestyle practices that are performed in the lifetime.^13-15^ One optimal resolution to reducing HTN is managing diet, especially by optimizing food sources available in the community.^16^ Of course, this problem needs to be resolved immediately so that people with HTN have the correct management in treating HTN.

 The government has managed HTN in the community through public health centers with a disease treatment process using community-based empowerment.^17^ Efforts that can be made include promotive and rehabilitative strategies.^18^ The Healthy Indonesia Program with a Family Approach, or in Indonesia called *Program Indonesia Sehat-Pendekatan Keluarga* (PIS-PK), is one of the programs implemented at the community health center level with a family approach.^19^ The program has excellent purposes, but the results of evaluations from various studies demonstrate that implementing PIS-PK as a form of community nursing care service still needs to be improved. This program can still not empower the family’s function in providing healthcare to individuals with health problems. Treating HTN at home depends more on those closest to you, such as family, than on health workers.

 The family has a vital role in treating HTN at home.^20^ Families of people with HTN also have a role in supervising the care management of people with HTN. The people with HTN lifestyle regarding meeting dietary needs (sodium consumption) also mainly involve the family preparing. Community nurses are essential in accommodating family empowerment in managing HTN.^21^ Family involvement as an approach in HTN management to maintain blood pressure (BP) and sodium diet compliance in people with HTN needs to be developed. This research is fundamental (urgent) because the family self-management program (FSMP) needs to be tested and evaluated for its success in managing HTN. Therefore, this research seeks to evaluate the effectiveness of FSMP in HTN management and adherence to sodium consumption in rural areas of Indonesia.

## Methods

###  Design

**Figure 1:**
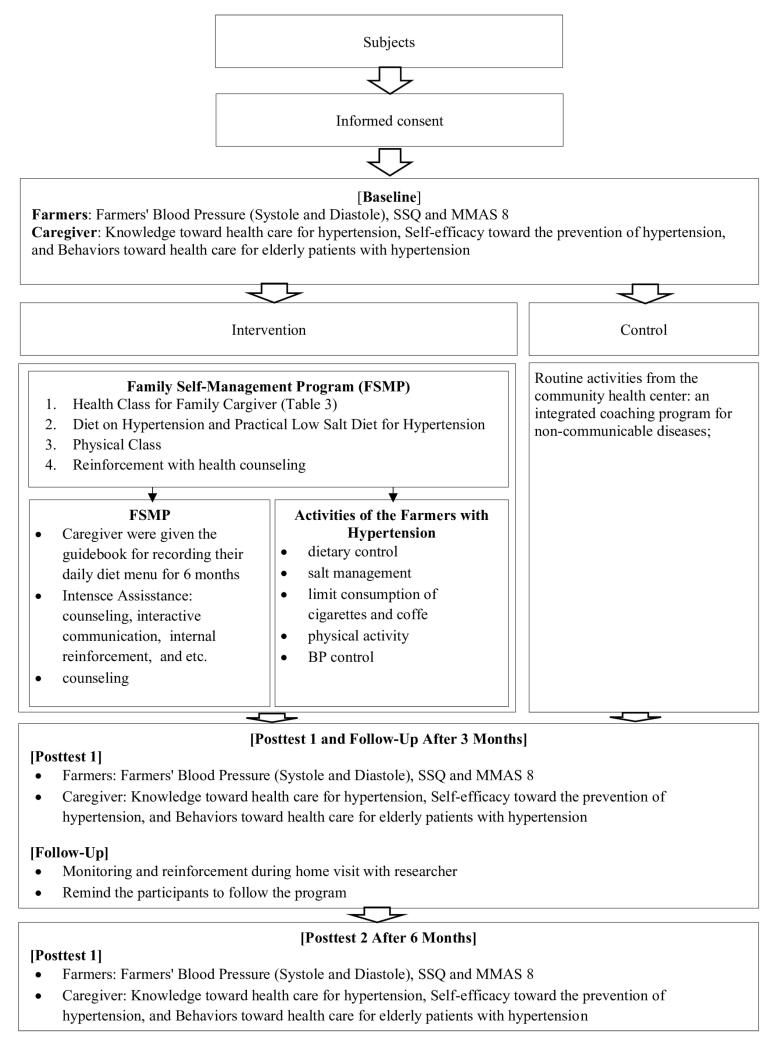
Procedure for Family Self-Management Program (FSMP)

 This study was a parallel randomized control trial based on community program interventions. This research was conducted in the Public Health Center Jember Regency, East Java, Indonesia, from April 2023 to October 2023. This investigation was located in four public health centers, including Banjarsengon, Sukorambi, Panti, and Pakusari, which were chosen because the number of people with HTN is quite significant in Jember Regency. Following the Consolidated Standards of Reporting Trials (CONSORT) statement, the participants were randomly assigned to the intervention (which received FMSP) or control group. Figure 1 shows the FSMP implemented in this study.

###  Sample and randomization

 The sample size required to evaluate the effectiveness of FSMP in managing HTN and adherence to sodium consumption in rural areas of Indonesia was calculated using the G*Power 3.1 program. The calculation used analysis of variance (ANOVA) repeated measures with an effect size of 0.25 and a pre-specified statistical power of 0.90. The number of groups and number of measurements were two and three, respectively. The estimated total sample size was 116 people with HTN. However, 126 participants were studied to account for a 10% attrition rate, divided into intervention and control groups.

 About 470 individuals with HTN from four public health centers in the Jember Regency (Banjarsengon, Sukorambi, Panti, and Pakusari) underwent screening to determine their eligibility for the study. The inclusion criteria involved individuals aged 45-65 years with stage I HTN, indicated by systolic BP (SBP) of 140-159 mmHg and diastolic BP (DBP) of 90-99 mmHg, as per the Indonesian Ministry of Health Guidelines (Indonesian Ministry of Health, 2021). Meanwhile, the exclusion criteria in this study were people with HTN who were not cared for by family members and were not willing to participate in the FSMP.

 Community health care professionals and community health workers in the research location performed the process of enrolling participants, randomization allocation, and assigning participants to interventions. One hundred twenty-six eligible participants were randomly allocated using a simple randomization method with computer-generated numbers. The participants were divided into the intervention group (INT, which received FMSP; n = 63) and the control group (CON, which received treatment as usual from a public health center: an integrated coaching program for NCDs; n = 63). Before starting the study, participants were required to sign an informed consent form after receiving both verbal and written information about the study procedure.

**Figure 2:**
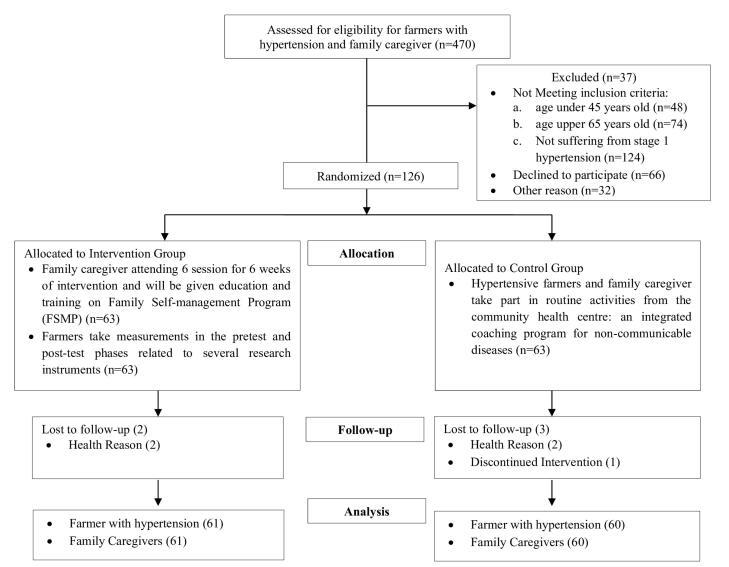
Consort Flow Diagram for enrolment, allocation, and follow-up study participants

 After 24 weeks of intervention, 2 participants in the intervention group discontinued as participants due to health reasons, and 3 participants in the control group discontinued as participants due to health and personal reasons. At the end of the intervention, the number of participants was 121 in the INT (n = 61) and CON (n = 60) groups. The selection of participants and the CONSORT flow diagram for enrollment, allocation, and follow-up study participants are provided in Figure 2.

###  Interventions

 This study was conducted for 24 weeks (6 months) with the FMSP intervention for family caregivers who cared for people with HTN. The FMSP intervention was performed over six sessions, each containing four series of health class educational activities, with the duration for each session being 60 minutes to 240 minutes. These health education classes included (1) a health class for family caregivers, (2) a diet on HTN and a practical low-salt diet for HTN, (3) a physical class, and (4) reinforcement with health counseling.

 The activities of people with HTN were evaluated by controlling diet, managing sodium intake, limiting cigarette and coffee consumption, doing physical activity, and measuring BP. According to the National Guidelines for Medical Services for the Management of Adult HTN, the daily limit for sodium and sodium chloride salt intake should not exceed 2 g/d (equivalent to 5-6 g of NaCl per day or one teaspoon of table salt).^22^ During the implementation of the FMSP intervention, people with HTN and caregivers were given a guideline module for six months, which was implemented from the initial stage, namely, the pre-intervention stage (baseline measurement); three months later, post-test one was performed, and finally follow-up or post-test 2 was conducted after six months. Meanwhile, the control group was given the treatment as usual by the Integrated NCD Development Post in Indonesia called *Pos Binaan Terpadu Penyakit Tidak Menular* from the Public Health Center in Jember. The respondents in this study, both intervention and control groups, were assessed at the beginning (baseline), follow-up 1/post-test 1 (after three months), and follow-up 2/post-test 2 (after six months).

###  Measures 

 The instruments used in the study consisted of three parts, namely, a demographics questionnaire, primary outcome measurements, and secondary outcome measurements. The first part was the demographic questionnaire of both people with HTN and family caregivers. For people with HTN, qualitative demographic characteristics consisted of gender, ethnicity, educational level, history of HTN, allergy, smoking, coffee consumption, sodium consumption, fat consumption, working hours, and rest during the week. The quantitative demographic characteristics of people with HTN included age, long hours of work in a week, working times in the weeks, abdominal circumference, waist circumference, height, weight, body mass index (BMI), and blood sugar. The family caregivers’ demographic characteristics encompassed gender, education level, marital status, occupation, income, relationship with the patient, alcohol consumption, active smoking, source of information about HTN, and age. This demographics questionnaire was then used to perform univariate and bivariate analysis to determine the frequency distribution of demographic characteristics and the relationship between the categories of questions in people with HTN and caregivers.^23^

 The primary outcomes of this study were SBP, DBP, the Score Sodium Questionnaire (SSQ), and the Morisky Medication Adherence Scale 8 (MMAS 8). BP measurements were performed by professional health workers at community health centers following the guidelines recommended by the Indonesian Ministry of Health (2021).^22^ The measurements were taken using an ABN Premium Aneroid sphygmomanometer designed for adults (ref: AS-011-LF), which has been validated for accuracy over 6-12 months. The measurement procedure involves the participant sitting comfortably and resting for 5 minutes prior to the examination. The Scored Sodium Questionnaire (SSQ) was given to people with HTN to measure the amount of sodium consumed over the past six months. This questionnaire comprises nine categories of questions with a total of 35 items of questions consisting of several questions categorized by type of feed, including (1) bread/nuts, (2) salad, (3) cereals, biscuits, and cakes, (4) cheese and savory, (5) Tinned and packed foods and other meal components, (6) meat and seafood dishes, (7) sweeteners &salt /flavor added in cooking, (8) sweeteners &salt /flavor added on table or salt, and (9) foods and drinks prepared from outside. The answers have four interpretations, starting from not eating, 1⁄2 serving a day, one serving per day, and more than two servings a day. If the participants choose the desired answer, it will be multiplied by 0 to 30, depending on the given question. The scores used in this questionnaire vary from 0 to a maximum of 215. The results are interpreted into two categories, namely, when ≥ 65, then high sodium consumption, and when ≤ 65, then low sodium intake. The validity and reliability of this questionnaire have been tested in previous studies and have been validated and reliable.^24^ In addition, MMAS 8 is a questionnaire that measures adherence levels in people with a history of HTN in evaluating and monitoring compliance in antihypertensive drug consumption. This questionnaire has been re-translated from English to Indonesian and has been adapted. The total number of questions in this questionnaire is eight, with a choice of answers, namely, “Yes” or “No”, with a scoring system of 1 on questions 1-3 and 5-8 when answering “NO” and a score of 1 for answers “YES” on question number 4. The minimum score range of these questionnaires is 0 to 8. The higher the scores imply, the higher the compliance respondents have with antihypertensive medication. The Indonesian version of the MMAS 8 questionnaire has demonstrated good reliability (Cronbach’s alpha 0.824) and validity (r = 0.883, sensitivity = 82.575%, specificity = 44.915%) in previous studies.^25-28^

 The secondary outcomes consisted of (1) knowledge of healthcare for HTN, (2) self-efficacy toward the prevention of HTN, and (3) behaviors toward healthcare for patients with HTN. The knowledge of healthcare for HTN questionnaire was given to the caregiver who cares for people with high BP. This questionnaire consists of 22 questions about healthcare for HTN. There are two answers from this knowledge questionnaire (“Yes” or “No”). In questions 4, 13,14,17,18, if the answer is “NO”, then it is given a score of 1, whereas in questions 1-12, 15-16, and 19-22, if the reply is “YES”, then the score is given 1.^29^ The self-efficacy questionnaire given to caregivers aims to measure the level of ability and willingness for prevention and control in caregivers who care for people with HTN. This questionnaire consists of 10 questions, with the type of question measuring the level of self-efficacy. The choice of answers in this study was disagree (scored 1), not sure (scored 2), and agree (scored 3). The self-efficacy questionnaire has a minimum score of 10 and a maximum of 30.^29^ The behavior toward healthcare for patients with HTN questionnaire was given to caregivers to measure caregiver behavior in performing healthcare in people with HTN. The monitoring in this questionnaire explains the dietary patterns of sodium consumption, lifestyle such as exercise or physical activity, sleep patterns, and beverages to be avoided. The number of questions from the following questionnaire is 20, with a choice of answers based on the four levels from never (scored 1), rarely (scored 2), sometimes (scored 3), to often (scored 4). The minimum and maximum scores are 20 and 80. The validity and reliability of this behavioral questionnaire have been tested on previously valid and reliable studies.^29^

###  Data analysis

 The gathered data were analyzed using SPSS 23.0 for Windows (SPSS Inc., Chicago, IL), and the study’s raw data were screened for accuracy and normality. A descriptive statistic was used for the characteristics of the respondents for qualitative variables such as gender, ethnicity, education level, history of HTN, allergic history, active smoking, coffee consumption, high salt consumption, fat consumption, work hours in a week, and rest periods during work. Quantitative variables included age, length of work hours, a workday in a week, abdominal circumference, waist circumference, height, weight, BMI, SBP, DBP, and random blood sugar. The Kolmogorov-Smirnov test was utilized to evaluate the distribution of quantitative variables. Normally and not normally distributed variables are presented as means and standard deviations (SD), as well as medians and interquartile ranges (P_25_-P_75_), respectively.

 The Chi-square test, Fisher’s exact test, and independent t-test were employed to measure different demographics of participants between the control and intervention groups before implementing the intervention, with statistical significance defined as *P* < 0.05. A between-group comparison using the independent t-test was performed on the variables related to people with HTN (SBP and DBP), SSQ, MMAS 8, caregivers’ knowledge, self-efficacy, and behaviors toward healthcare for people with HTN to analyze the differences of the FSMP program on the baseline, third month, and sixth month. ANOVA was used to assess the effectiveness of FSMP for managing BP and adherence to sodium consumption for people with HTN. The effect size was included to support relevant findings and was examined using Cohen’s d for between-group differences (0.2: small, 0.5: medium, and 0.8: large effects).^30^

###  Ethical considerations 

 This study considers ethical feasibility to be an essential aspect of research. The research participants had the right to either participate or not participate in the research process, provide verbal and written consent, and observe the principle of information confidentiality of the respondents participating in the study. This research was approved by the Research Ethics Committee of the Faculty of Dentistry, University of Jember No. 2044/UN25.8/KEPK/DL/2023.

## Results

 The data collection process, including recruitment, screening, baseline measurements, and interventions, was completed between April and June 2023. The follow-up 1/post-test 1, and follow-up 2/post-test 2 were conducted in July 2023 and October 2023, respectively.The results of this study are presented in Tables 1 and 2. Table 1 presents the INT and CONT groups’ people with HTN demographic characteristics and homogeneity test results. In both groups, gender was mainly female (INT = 70.3%, CONT = 78.3%), with an average age (INT = 54.89, CONT = 53.75) of Madurese ethnicity (INT = 75.3%, CONT = 71.7%), and all were Muslim (100%). The respondents’ education level distribution showed that most had elementary school education (INT = 59%, CONT = 48.3%), and the least had a bachelor’s degree program. More than half of all respondents experienced HTN for more than five years (INT = 77%, CONT = 80%) and were in the Grade I HTN with an average SBP (INT = 147.62, CONT = 146.37) and DBP (INT = 93.64, CONT = 89.28). The history of allergies indicated that the majority of respondents had no allergies (INT = 95.1%, CONT = 91.7%) and did not actively smoke (INT = 80.3%, CONT = 86.7%). The results of the homogeneity analysis revealed that the *P* was > 0.05, implying that the two groups’ demographic variables were homogeneous.

**Table 1:** Comparison of demographic information in the two groups at the pre-intervention phase on people with hypertension and family caregiver

**Variables**	**Intervention group (n=61)**		**Control group (n=60)**		
	**Number**	**Percent**	**Number**	**Percent**	***P *** **value**
Gender					
Male	18	29.5	13	21.7	0.325
Female	43	70.3	47	78.3	
Ethnic					
Java	15	24.6	17	28.3	0.642
Madura	46	75.3	43	71.7	
Education level					
Not attending school	16	26.2	12	20.0	0.056
Elementary school	36	59.0	29	48.3	
Junior high school	7	11.5	12	20.0	
Senior high school	1	1.6	6	10.0	
Bachelor program	1	1.6	1	1.7	
History of hypertension					
Less than 5 years	47	77.0	48	80.0	0.694
More than 5 years	14	23.0	12	20.0	
Allergic history					
Yes	3	4.9	5	8.3	0.452
No	58	95.1	55	91.7	
Active smoking					
Yes	12	19.7	8	13.3	0.350
No	49	80.3	52	86.7	
Coffee consumption					
Yes	30	49.2	30	50.0	0.928
No	31	50.8	30	50.0	
High salt consumption					
Yes	41	67.2	20	33.3	0.949
No	20	32.8	40	66.7	
Fat consumption					
Yes	20	32.8	31	51.7	0.083
No	41	67.2	29	48.3	
Work in a week (h)					
≤ 40	23	37.7	20	33.3	0.617
> 40	38	62.3	40	66.7	
Rest period during work (min/d)					
< 30	2	3.3	3	5.0	0.365
> 30	59	96.7	57	95.0	
	**Mean**	**SD**	**Mean**	**SD**	***P *** **value**
Age (y)	54.89	5.91	53.75	6.33	0.169
Length of work hour in a week	45.00	17.59	44.8	17.97	0.729
Working time, a week	6.43	1.18	6.88	0.42	0.004
Abdominal circumference	87.57	12.48	88.58	12.11	0.595
Waist circumference	96.70	12.92	95.92	11.63	0.732
Height	154.59	7.32	152.70	4.95	0.093
Weight	58.15	12.55	58.00	11.08	0.963
Body mass index	24.22	5.13	24.84	4.52	0.302
Systolic blood pressure	147.62	14.23	146.37	15.09	0.823
Diastolic blood pressure	93.64	13.89	89.28	15.09	0.651
Blood sugar	121.25	35.21	126.53	74.62	0.272
Family caregiver					
	**Number**	**Percentage**	**Number**	**Percentage**	***P *** **value**
Gender					
Male	25	41.0	35	58.3	0.062
Female	36	59.0	25	41.7	
Education level					
Not attending school	11	18.0	9	15.0	0.522
Elementary school	26	42.6	41	68.3	
Junior high school	19	31.1	9	15.0	
Senior high school	3	4.9	1	1.7	
Bachelor program	2	3.3	0	0.0	
Marital status					
Single	7	11.5	2	3.3	0.813
Married	53	86.9	58	96.7	
Divorced	0	0.0	0	0.0	
Separated	0	0.0	0	0.0	
Widowed	1	1.6	0	0.0	
Occupation					
Unemployment	18	29.5	9	15.0	0.061
Employee	1	1.6	1	1.7	
Agriculture sector	26	42.6	26	43.3	
Merchant	11	18.0	23	38.3	
Others	5	8.2	1	1.7	
Income					
Enough	51	83.6	54	90.0	0.211
Not enough	10	16.4	5	8.3	
Relation with patient					
Partner	35	57.4	50	83.3	0.082
Child	21	34.4	9	15.0	
Grandchild	3	4.9	1	1.7	
Others	2	3.3	0	0.0	
Alcohol consumption					
Ye	0	0.0	0	0.0	0.583
No	61	100.0	60	100.0	
Active smoking					
Yes	11	18.0	16	26.7	0.442
No	50	82.0	44	73.3	
Get Education about hypertension					
No	13	21.3	29	48.3	0.232
Yes, by health worker	16	26.2	8	13.3	
Yes, by health volunteer	28	45.9	23	38.3	
Yes, by online media	2	3.3	0	0.0	
Yes, by others	2	3.3	0	0.0	
	**Mean**	**SD**	**Mean**	**SD**	***P *** **value**
Age (year)	47.21	13.37	48.23	12.71	0.094

*Note*. SD: Standard deviation.

**Table 2:** Repeated measures ANOVA of people with hypertension and caregiver outcomes between the intervention and control groups

**Groups**	**Baseline**		**After 3 Months**		**After 6 Months**		***P*** ** value**	**Partial Eta** **squared**	**Effect** **size**
**Variables**	**Mean**	**SD**	**Mean**	**SD**	**Mean**	**SD**			
People with systolic hypertension									
Intervention	147.62	14.23	146.97	11.22	131.46	10.62	0.004	0.11	0.352
Control	146.37	15.09	146.25	15.23	145.38	14.33			
*P*-between	0.815		0.211		0.022				
People with diastolic hypertension									
Intervention	93.64	13.89	91.51	10.28	84.08	9.31	0.006	0.32	0.686
Control	89.28	15.09	89.55	14.41	90.77	13.91			
*P*-between	0.648		0.094		0.001				
Score Sodium Questionnaire									
Intervention	71.96	8.76	67.97	6.56	42.11	5.76	0.001	0.374	0.773
Control	71.82	7.52	72.59	8.32	71.16	8.21			
*P*-between	0.966		0.021		0.001				
Morisky Medication Adherence Scale 8									
Intervention	6.43	1.32	7.02	1.21	7.56	1.32	0.001	0.391	0.801
Control	6.72	1.02	6.60	1.11	6.37	1.27			
*P*-between	0.468		0.082		0.031				
Caregivers’ knowledge									
Intervention	17.87	5.23	19.62	6.12	21.36	4.33	0.001	0.252	0.58
Control	17.57	4.21	17.73	5.87	17.85	4.69			
*P*-between	0.595		0.034		0.002				
Caregivers’ self-efficacy									
Intervention	26.62	3.21	27.49	3.89	29.34	2.32	0.001	0.189	0.483
Control	26.70	5.58	25.91	4.21	26.02	4.19			
*P*-between	0.894		0.022		0.013				
Caregivers’ behaviours									
Intervention	58.30	6.12	57.03	5.89	71.23	5.12	0.005	0.566	1.141
Control	58.42	5.18	59.05	5.46	59.38	5.99			
*P*-between	0.641		0.053		0.036				

*Note*. ANOVA: Analysis of variance; SD: Standard deviation.

 As regards demographic data on family caregivers from the intervention and control groups, it was found that the majority of participants were female (INT = 59%, CONT = 41.7%), with an average age of over 45 years (INT = 47.21, CONT = 48.23). Most had elementary school-level education (INT = 42.6%, CONT = 68.3%). The majority were married (INT = 86.9%, CONT = 96.7%), worked in the agricultural sector (INT = 42.6%, CONT = 43.3%), and earned sufficient income (INT = 83.6%, CONT = 90%). Most of them were spouses of people with HTN (INT = 57.4%, CONT = 41.7%) (70.2%). None of the respondents consumed alcohol (100%), and most were not active smokers (INT = 82%, CONT = 73.3%).

 The analysis between the two groups in each measurement phase was also performed using the independent t-test (Table 2). The variables analyzed included people with HTN BP (SBP and DBP), SSQ, MMAS 8, caregivers’ knowledge, self-efficacy, and behaviors toward healthcare for people with HTN. The analysis demonstrated that almost all variables had significant differences (*P* < 0.05) between the INT and CONT groups at measurements three and six months after the implementation of FSMP. Nonetheless, regarding the SBP variable, after three months of therapy, no significant difference was found between the INT and CONT groups (*P* > 0.05). However, it changed in the third measurement of the six-session FSMP intervention (Table 3).

**Table 3:** Family self-management program for management of hypertension and adherence to sodium consumption

**Sessions**	**Objectives**	**A summary of topics and activities**	**Educational time (min)**
1	Hypertension disease concept	Identifying the causes of hypertension and changes in lifestyle as well as unhealthy diet patterns, leading to the risk of high blood pressure.	60
2	The role of the family in caring for a sick family member	Explaining the role of the family in the care of sick family members and its responsibilities in treating sick families.	60
3	Family responsibilities in caring for sick family members	Focusing on the tasks that the family can do as the primary supporting component in supporting dietary pattern management as well as medication for hypertension.	60
4	Hypertension diet in family order	Introducing restrictions on sodium intake through the type of food or drink consumed to be avoided or reduced as a form of family treatment for patients with hypertension with the Dietary Approaches to Stop Hypertension (DASH) Eating Plan guide.	90
5	Practical low-salt diets for hypertensive patients	Training families with hypertensive patients in introducing low-salt diets using the DASH Eating Plan.	240
6	Low-salt diet and physical activity for hypertensive patients	Simulating a menu of household foods with a low-salt diet according to the DASH Eating Plan and a variety of physical activities that can be performed as a supportive form in preventing hypertension.	240

**Figure 3:**
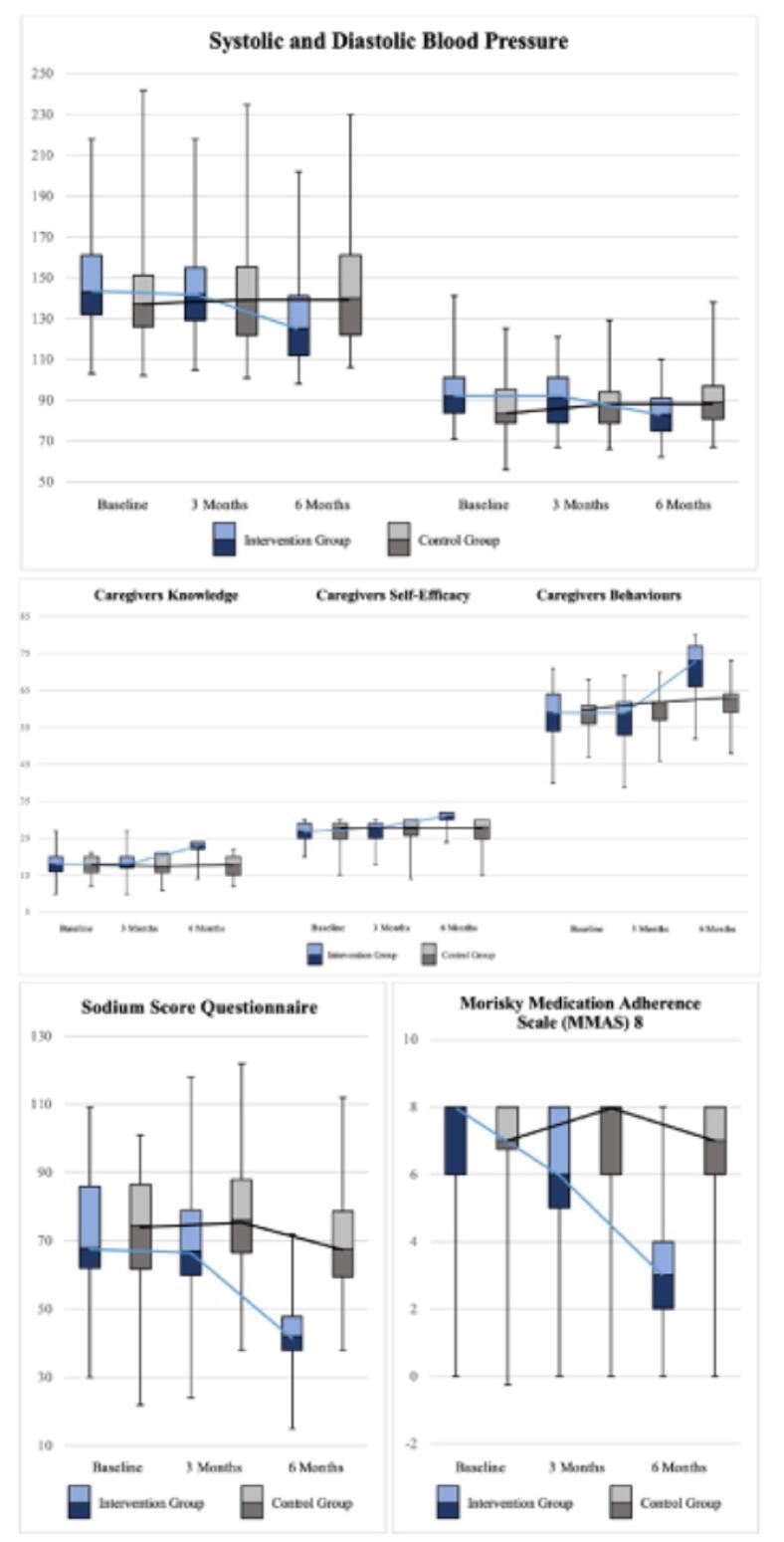
Boxplot of the effects of the Family Self-Management Program

 Using the ANOVA test, the analysis generally indicated that the FSMP performed on farmers with HTN and family caregivers provided significant changes in all variables measured in this study (*P*< 0.05). These results suggest that the FSMP program in people with HTN and their family caregivers can help control BP (SBP and DBS) and compliance with dietary sodium. Furthermore, Cohen’s d analysis revealed a range of effect sizes, from small (d = 0.352) to large (d = 8.01), for primary outcomes. Similarly, the analysis also showed effect sizes ranging from small (0.483) to large (1.141) for the secondary outcomes. The detailed analysis of the effect of FMSP on BP, SSQ, MMAS 8, caregivers’ knowledge, self-efficacy, and behaviors is illustrated in Figure 3.

## Discussion

 The findings of this study confirmed that the FSMP was effective in managing HTN, improving adherence to sodium consumption among people with HTN, and enhancing family caregivers’ knowledge of healthcare, self-efficacy, and behavior toward patients with HTN healthcare. The FMSP promotes reduced BP (systolic and diastolic), decreased salt consumption, and compliance with antihypertensive medication. During the 24-week follow-up period, there were significant improvements in all variables studied about managing HTN in the intervention group compared to the control group.

 Caregivers of people with HTN have an essential role in ensuring adequate care. The results of this study demonstrated a significant improvement in knowledge about healthcare for HTN, self-efficacy toward the prevention of HTN, and behaviors toward healthcare for people with HTN, which were better in the intervention group than the control group after administering FMSP. One of the explanations for this improvement can be attributed to the health classes performed for caregivers, emphasizing the concept of HTN. At the same time, this program concentrates on empowering caregivers to care for people with HTN by clarifying the role of caregivers in caring for sick family members and the caregiver’s responsibilities as a primary supporting component for people with HTN. Specific education programs for caregivers can diminish gaps in caregiver knowledge and raise awareness and care practices; they can also improve the quality of life, ultimately enhancing their caregiving role.^31^ The family is a source of information for patients, and family caregivers tend to summarize health information provided by healthcare providers, provide it briefly, and filter it according to people with HTN preferences so that it does not drive pressure.^32^ Previous research indicated that health education can enhance knowledge and attitudes in HTN management and treatment compliance, which can be improved with regular follow-ups.^33,34^ Self-efficacy is an individual’s belief in their capacity to perform behavior that results in specific achievements. Higher self-efficacy relates to self-care management and involvement in self-care behavior.^35,36^ This study’s results conform to those of previous research, which showed that family training programs can increase knowledge, self-efficacy, and behavior in health services and strengthen the relationship between family and people with HTN in self-care.^29^ Self-efficacy, along with support from family, increases health-promoting HTN behavior.^37^

 In this study, the mean score of the SSQ that measures the amount of sodium consumed by people with HTN over the past six months confirmed a decline, demonstrating that the dietary habits of people with HTN are related to the results of FMSP implementation. People with HTN and caregivers participate in HTN diet management and the practice of a low-salt diet for HTN through efforts such as diet control, salt management for people with HTN, and a guidebook for recording daily diet menus for caregivers. Research results revealed that collaboration between patients, families, and professional health workers positively impacts self-management behavior in chronic disease.^38-40^ Simultaneously, caregivers help recognize unhealthy processed foods and can limit their consumption, which is also influenced by salt-related knowledge, attitude, and behavior.^41-43^ In addition, dietary patterns contribute to BP, where dietary interventions consisting of dietary patterns and dietary management play an important role in managing HTN through the dietary approach to stop HTN,^44,45^ as in this study, a book was used to document the daily diet, including diet patterns and dietary arrangements, for people with HTN.

 The level of compliance with antihypertensive medication (MMAS) improved after participating in FMSP. One of the reasons for this enlargement is correlated to intensive assistance, which includes communication, internal strengthening, and health counseling by health providers. Family support is an important component in fulfilling treatment because the family provides motivation and is directly involved in preparing medication.^46^ Further, family involvement in patient education significantly enhances treatment adherence and outcomes in HTN through encouragement for healthy lifestyle changes and better BP management.^47^ Counseling, also part of the FMSP, provides optimal health education by increasing trust, family support, and motivation.^48^ Education impacts health literacy;, in turn, health literacy, together with social support, especially from the family, directly influences fulfilling HTN treatment.^49-51^

 The decrease in hypertensive BP (systolic and diastolic) indicates that FMSP produces good outcomes in people with HTN. Moreover, caregiver involvement promotes and strengthens the self-regulation of people with HTN, which results in controlled BP. Furthermore, BP that declines significantly is related to people with HTN independent activities of HTN, including controlling diet, lowering consuming coffee and cigarettes, engaging in physical activity, and controlling BP. The findings of this study are in line with those of previous investigations, which demonstrated that the critical role of caregivers in providing social support, caring participation, communicating health, and better self-care behavior for people with HTN can reduce BP.^29^ BP can be effectively controlled with self-management interventions,^52^ which include self-regulation, self-monitoring, and treatment adherence (monitoring BP at home, limiting salt intake, and exercise).^53,54^ Family support programs can improve the quality of care for chronic illnesses,^55^ including physical, psychological, and financial capabilities. In conclusion, the FMSP can effectively reduce BP in people with HTN through the support of family caregivers and self-management activities.

 Family support programs can improve the quality of care for chronic illnesses,55 including physical, psychological, and financial capabilities. Furthermore, addressing lifestyle factors such as sodium consumption can lead to an improved quality of life for individuals with hypertension.^56^ In conclusion, the FMSP can effectively reduce BP in people with HTN through the support of family caregivers and self-management activities.

 The limitation of this research was that the participants needed help writing and were assisted in filling out the dietary care workbook. However, all participants can complete the research stages and finish the guidebook, thus minimizing bias in the research results. This study also did not perform a multivariate analysis to identify factors related to the management of HTN and adherence to sodium consumption. Further research is needed to explore this issue. In addition, this research was conducted in the Jember Regency, East Java, Indonesia, an agricultural area; thus, the research results may not represent other populations but can be applied to areas with similar characteristics.

**Highlights**

- The high incidence of hypertension (HTN), which is a non-communicable disease (NCD) problem, requires treatment management with different strategies.
- Families of people with HTN also have a role in supervising the care management of people with HTN.
- The family self-management program (FSMP) implemented to maintain blood pressure (BP) and adherence to sodium consumption among farmers could significantly influence farmers’ BP.
- The FMSP emerges as a promising strategy for reducing BP in people with HTN through the support of family caregivers and self-management activities.

## Conclusion

 The findings indicated that the FSMP was implemented to maintain the management of HTN and adherence to sodium consumption among people with HTN. Notably, the FMSP intervention significantly influenced people with HTN BP (SBP and DBP), SSQ, MMAS 8, and caregivers’ knowledge, self-efficacy, and behaviors toward healthcare over six months. It can be concluded that FSMP interventions can promote and significantly improve BP and adherence to sodium consumption among people with HTN. The study contributes valuable insights into the potential benefits of this management HTN strategy for people with HTN, caregivers, and healthcare providers for health promotion activities for HTN patients and family members in the community.

## Acknowledgments

 The authors would like to express their deepest appreciation to the Faculty of Nursing of the University of Jember, Institute for Research and Community Service, Universitas Jember, the nurses in the public health centers in Jember, and the farmers and family caregivers in the rural areas of Jember Regency, Indonesia, for their invaluable support and contribution to the completion of this research.

## Authors’ Contribution

**Conceptualization:** Tantut Susanto and Sri Hernawati.

**Data curation:** Rismawan Adi Yunanto, Ira Rahmawati, and Niken Asih Laras Ati.

**Formal analysis:** Rismawan Adi Yunanto and Niken Asih Laras Ati.

**Funding acquisition:** Tantut Susanto.

**Investigation:** Tantut Susanto, Sri Hernawati, and Ira Rahmawati.

**Methodology:** Tantut Susanto, Rismawan Adi Yunanto, and Niken Asih Laras Ati.

**Project administration:** Ira Rahmawati and Sri Hernawati.

**Resources:** Tantut Susanto and Sri Hernawati.

**Software:** Ira Rahmawati and Sri Hernawati.

**Supervision:** Tantut Susanto.

**Validation:** Sri Hernawati and Ira Rahmawati.

**Visualization:** Rismawan Adi Yunanto and Niken Asih Laras Ati.

**Writing–original draft:** Tantut Susanto, Rismawan Adi Yunanto, Ira Rahmawati, Sri Hernawati, Niken Asih Laras Ati, and Wahyuni Fauziah.

**Writing–review & editing:** Niken Asih Laras Ati, Tantut Susanto, Rismawan Adi Yunanto, and Wahyuni Fauziah.

## Competing Interests

 The authors declare no conflict of interests. All authors have read and agreed to the published version of the manuscript.

## Ethical Approval

 Ethical approval was granted for this study. The Research Ethics Commission of the Faculty of Dentistry, University of Jember (No. 2044/UN25.8/KEPK/DL/2023) approved the study. The authors declare that the present manuscript has not been published or submitted to any other journal.

## Funding

 This research was financially supported by Grant Applied Research of Professor Productivity (Grant number 3540/UN25.3.1/LT/2023), Institute for Research and Community Service, Universitas Jember.

## References

[1] World Health Organization (WHO). Noncommunicable Diseases. Fact and Sheets. WHO; 2023. Available from: https://www.who.int/news-room/fact-sheets/detail/noncommunicable-diseases. Accessed February 3, 2024.

[2] Mills KT, Stefanescu A, He J (2020). The global epidemiology of hypertension. Nat Rev Nephrol.

[3] Kementerian Kesehatan Republik Indonesia. Hasil Utama RISKEDAS 2018. Jakarta: Kementerian Kesehatan Republik Indonesia; 2018. Available from: https://www.kemkes.go.id/resources/download/info-terkini/hasil-riskesdas-2018.pdf. Accessed September 20, 2023.

[4] As’ady BA, Supangat S, Indreswari L (2019). Analysis of personal protective equipments pesticides usage effects on health complaints of farmers in Pringgondani Village Sumberjambe District Jember Regency. Journal of Agromedicine and Medical Sciences.

[5] Aristi DL, Rasni H, Susumaningrum LA, Susanto T, Siswoyo S (2020). The relationship between high sodium food consumption and the incidence of hypertension among farm workers at public health centre of Panti in Jember Regency. Buletin Penelitian Sistem Kesehatan.

[6] Susanto T, Purwandari R, Wuryaningsih EW (2016). Occupational health nursing model-based agricultural nursing: a study analyzes of farmers health problem. Jurnal Ners.

[7] Rusmevichientong P, Morales C, Castorena G, Sapbamrer R, Seesen M, Siviroj P (2021). Dietary salt-related determinants of hypertension in rural northern Thailand. Int J Environ Res Public Health.

[8] Subasinghe AK, Arabshahi S, Busingye D, Evans RG, Walker KZ, Riddell MA (2016). Association between salt and hypertension in rural and urban populations of low to middle income countries: a systematic review and meta-analysis of population-based studies. Asia Pac J Clin Nutr.

[9] Ghimire K, Adhikari TB, Rijal A, Kallestrup P, Henry ME, Neupane D (2019). Knowledge, attitudes, and practices related to salt consumption in Nepal: findings from the community-based management of non-communicable diseases project in Nepal (COBIN). J Clin Hypertens (Greenwich).

[10] Astutik E, Puspikawati SI, Dewi D, Mandagi AM, Sebayang SK (2020). Prevalence and risk factors of high blood pressure among adults in Banyuwangi coastal communities, Indonesia. Ethiop J Health Sci.

[11] McKenzie B, Santos JA, Trieu K, Thout SR, Johnson C, Arcand J (2018). The Science of Salt: a focused review on salt-related knowledge, attitudes and behaviors, and gender differences. J Clin Hypertens (Greenwich).

[12] Ndanuko RN, Tapsell LC, Charlton KE, Neale EP, Batterham MJ (2016). Dietary patterns and blood pressure in adults: a systematic review and meta-analysis of randomized controlled trials. Adv Nutr.

[13] Abu H, Aboumatar H, Carson KA, Goldberg R, Cooper LA (2018). Hypertension knowledge, heart healthy lifestyle practices and medication adherence among adults with hypertension. Eur J Pers Cent Healthc.

[14] Sabouhi F, Babaee S, Naji H, Hassan Zadeh A (2011). Knowledge, awareness, attitudes and practice about hypertension in hypertensive patients referring to public health care centers in Khoor & Biabanak. Iran J Nurs Midwifery Res.

[15] Yang L, Xu X, Yan J, Yu W, Tang X, Wu H (2014). Analysis on associated factors of uncontrolled hypertension among elderly hypertensive patients in Southern China: a community-based, cross-sectional survey. BMC Public Health.

[16] Susanto T, Rasny H, Kurdi F, Yunanto RA, Rahmawati I (2023). Management of hypertension using a plant-based diet among farmers: protocol for a mixed methods study. JMIR Res Protoc.

[17] Direktorat P2PTM Ditjen Pencegahan dan Pengendalian Penyakit. Manajemen Program Pencegahan dan Pengendalian Hipertensi dan Perhitungan Pencapaian SPM Hipertensi. Bali; 2018.

[18] Vandiver T, Anderson T, Boston B, Bowers C, Hall N (2018). Community-based home health programs and chronic disease: synthesis of the literature. Prof Case Manag.

[19] Fauzi R, Efendi R, Mustakim M (2020). Program Pengelolaan Penyakit Hipertensi Berbasis Masyarakat dengan Pendekatan Keluarga di Kelurahan Pondok Jaya, Tangerang Selatan. Wikrama Parahita: Jurnal Pengabdian Masyarakat.

[20] Bahari G, Scafide K, Krall J, Mallinson RK, Weinstein AA (2019). Mediating role of self-efficacy in the relationship between family social support and hypertension self-care behaviours: a cross-sectional study of Saudi men with hypertension. Int J Nurs Pract.

[21] Grady PA, Gough LL (2014). Self-management: a comprehensive approach to management of chronic conditions. Am J Public Health.

[22] Indonesian Ministry of Health. National Guidelines for Medical Services for the Management of Adult Hypertension. 2021. Report No.: HK.01.07/MENKES/4634/2021.

[23] Susanto T, Rasny H, Susumaningrum LA, Yunanto RA, Nur KR (2019). Prevalence of hypertension and predictive factors of self-efficacy among elderly people with hypertension in institutional-based rehabilitation in Indonesia. Kontakt.

[24] Wong ATY, Munt A, Allman-Farinelli M, Badve SV, Boudville N, Coolican H (2020). Assessment of dietary sodium intake using the scored salt questionnaire in autosomal dominant polycystic kidney disease. Nutrients.

[25] Riani DA, Ikawati. Z, Kristina SA. Validation of the 8-Item Morisky Medication Adherence Scale Indonesian Version in Adult Hypertension Patients in the Public Health Center of Sleman and Yogyakarta City [thesis]. Universitas Gadjah Mada; 2017.

[26] Vera MO, Susilowati E (2019). Compliance of hypertension patients in drinking drugs in Pakis district, Malang Regency. Akademi Farmasi Putera Indonesia Malang.

[27] Zhang Y, Wang R, Chen Q, Dong S, Guo X, Feng Z (2021). Reliability and validity of a modified 8-item Morisky Medication Adherence Scale in patients with chronic pain. Ann Palliat Med.

[28] Martinez-Perez P, Orozco-Beltrán D, Pomares-Gomez F, Hernández-Rizo JL, Borras-Gallen A, Gil-Guillen VF (2021). Validation and psychometric properties of the 8-item Morisky Medication Adherence Scale (MMAS-8) in type 2 diabetes patients in Spain. Aten Primaria.

[29] Boonyathee S, Seangpraw K, Ong-Artborirak P, Auttama N, Tonchoy P, Kantow S (2021). Effects of a social support family caregiver training program on changing blood pressure and lipid levels among elderly at risk of hypertension in a northern Thai community. PLoS One.

[30] Lakens D (2013). Calculating and reporting effect sizes to facilitate cumulative science: a practical primer for t-tests and ANOVAs. Front Psychol.

[31] Nath SD, Chowdhury AS, Pinky SD, Akter KM, Nourin NA, Chowdhury T (2023). Covariates of knowledge, attitude, practice, and burdens among the caregivers of hypertensive patients. Int J Hypertens.

[32] Al-Ananbeh E, Al-Wahadneh A (2020). Experiences of family caregivers’ involvement in treatment related-decision-making in triadic health encounters. J Sci Res Med Biol Sci.

[33] Kurnia AD, Melizza N, Ruhyanudin F, Masruroh NL, Prasetyo YB, Setyowati CI (2022). The effect of educational program on hypertension management toward knowledge and attitude among uncontrolled hypertension patients in rural area of Indonesia. Community Health Equity Res Policy.

[34] Tolley A, Hassan R, Sanghera R, Grewal K, Kong R, Sodhi B (2023). Interventions to promote medication adherence for chronic diseases in India: a systematic review. Front Public Health.

[35] Tan FC, Oka P, Dambha-Miller H, Tan NC (2021). The association between self-efficacy and self-care in essential hypertension: a systematic review. BMC Fam Pract.

[36] Zhang Q, Huang F, Zhang L, Li S, Zhang J (2021). The effect of high blood pressure-health literacy, self-management behavior, self-efficacy and social support on the health-related quality of life of Kazakh hypertension patients in a low-income rural area of China: a structural equation model. BMC Public Health.

[37] Giena VP, Thongpat S, Nitirat P (2018). Predictors of health-promoting behaviour among older adults with hypertension in Indonesia. Int J Nurs Sci.

[38] Aklima A, Kritpracha C, Thaniwattananon P (2012). Development of family-based dietary self-management support program on dietary behaviors in patients with type 2 diabetes mellitus in Indonesia: a literature review. Nurse Media J Nurs.

[39] Maneesri S, Masingboon K, Chaimongkol N (2023). Effectiveness of individual and family self-management combined mHealth program for people with stage 3 chronic kidney disease: a randomized controlled trial. Pac Rim Int J Nurs Res Thail.

[40] Rosland AM, Heisler M, Choi HJ, Silveira MJ, Piette JD (2010). Family influences on self-management among functionally independent adults with diabetes or heart failure: do family members hinder as much as they help?. Chronic Illn.

[41] Hoeft KS, Guerra C, Gonzalez-Vargas MJ, Barker JC (2015). Rural Latino caregivers’ beliefs and behaviors around their children’s salt consumption. Appetite.

[42] Grimes CA, Khokhar D, Bolton KA, Trieu K, Potter J, Davidson C (2020). Salt-related knowledge, attitudes and behaviors (KABs) among Victorian adults following 22-months of a consumer awareness campaign. Nutrients.

[43] Khokhar D, Nowson C, Margerison C, Bolam B, Grimes C (2019). Comparison of salt-related knowledge, attitudes and behaviours between parents and caregivers of children under 18 years of age and other adults who do not care for children under 18 years of age in Victoria, Australia. BMJ Nutr Prev Health.

[44] Margerison C, Riddell LJ, McNaughton SA, Nowson CA (2020). Associations between dietary patterns and blood pressure in a sample of Australian adults. Nutr J.

[45] Chang HC, Cheng HM, Chen CH, Wang TD, Soenarta AA, Turana Y (2021). Dietary intervention for the management of hypertension in Asia. J Clin Hypertens (Greenwich).

[46] Wiedyaningsih C, Widyakusuma NN, Suryawati S (2023). The role of family caregivers in medication adherence of elderly in Asian setting: a scoping review. Indones J Pharmacol Ther.

[47] Maslakpak MH, Rezaei B, Parizad N (2018). Does family involvement in patient education improve hypertension management? A single-blind randomized, parallel group, controlled trial. Cogent Med.

[48] Prihanti GS, Sari NP, Septiani NI, Tobing LP, Adrian AR, Ayu N (2020). The effect of counseling on the adherence of therapeutic hypertension patients. Jurnal Keperawatan.

[49] Guo A, Jin H, Mao J, Zhu W, Zhou Y, Ge X (2023). Impact of health literacy and social support on medication adherence in patients with hypertension: a cross-sectional community-based study. BMC Cardiovasc Disord.

[50] Pan J, Hu B, Wu L, Li Y (2021). The effect of social support on treatment adherence in hypertension in China. Patient Prefer Adherence.

[51] Shahin W, Kennedy GA, Stupans I (2021). The association between social support and medication adherence in patients with hypertension: a systematic review. Pharm Pract (Granada).

[52] Li R, Liang N, Bu F, Hesketh T (2020). The effectiveness of self-management of hypertension in adults using mobile health: systematic review and meta-analysis. JMIR Mhealth Uhealth.

[53] AlHadlaq RK, Swarelzahab MM, AlSaad SZ, AlHadlaq AK, Almasari SM, Alsuwayt SS (2019). Factors affecting self-management of hypertensive patients attending family medicine clinics in Riyadh, Saudi Arabia. J Family Med Prim Care.

[54] Omoronyia OE, Okesiji I, Uwalaka CH, Mpama EA (2021). Reported self-management of hypertension among adult hypertensive patients in a developing country: a cross-sectional study in a Nigerian tertiary hospital. Afr Health Sci.

[55] Vinsur EY, Handini FS, Nurwiyono A (2023). Family caregiver support program to increase quality care among the geriatric population. Jurnal Keperawatan Komprehensif (Comprehensive Nursing Journal).

[56] Pangestu AW, Kurdi F, Rasni H. The Relation Between Life Style and Quality of Life on Hypertension Farmers in Panti District, Jember Regency. J Rural Community Nurs Pract. 2024; 2(1):97–111. 10.58545/jrcnp.v2i1.226.

